# A pilot study of angiogenin in heart failure with preserved ejection fraction: a novel potential biomarker for diagnosis and prognosis?

**DOI:** 10.1111/jcmm.12344

**Published:** 2014-08-15

**Authors:** Hong Jiang, Lei Zhang, Ying Yu, Ming Liu, Xuejuan Jin, Peipei Zhang, Peng Yu, Shuning Zhang, Hongmin Zhu, Ruizhen Chen, Yunzeng Zou, Junbo Ge

**Affiliations:** Shanghai Institute of Cardiovascular Diseases, Zhongshan Hospital, Shanghai Medical College of Fudan UniversityShanghai, China

**Keywords:** heart failure with preserved ejection fraction, angiogenin, biomarker, proteomics

## Abstract

Characteristics of heart failure with preserved ejection fraction (HFPEF) have not yet been fully understood. The objectives of this pilot study are to detect protein expression profile in the sera of HFPEF patients, and to identify potential biomarkers for the disease. Five hundred and seven proteins were detected in the sera of healthy volunteers and patients with either HFPEF or hypertension using antibody microarrays (three in each group). The results showed that the serum concentrations of 17 proteins (*e.g*. angiogenin, activin A and artemin) differed considerably between HFPEF and non-HFPEF patients (hypertensive patients and healthy controls), while a protein expression pattern distinct from that in non-HFPEF patients was associated with HFPEF patients. The up-regulation of angiogenin in both HFPEF patients with LVEF ≥50% (*P* = 0.004) and a subset of HFPEF patients with LVEF = 41–49% (*P* < 0.001) was further validated in 16 HFPEF patients and 16 healthy controls. Meanwhile, angiogenin distinguished HFPEF patients from controls with a mean area under the receiver operating characteristic curve of 0.88 (*P* < 0.001) and a diagnostic cut-off point of 426 ng/ml. Moreover, the angiogenin levels in HFPEF patients were positively correlated with Lg(N-terminal pro-B-type natriuretic peptide, NT-proBNP) (*P* < 0.001). In addition, high angiogenin level (≥426 ng/ml) was a predictor of all-cause death within a short-term follow-up duration, but not in the longer term of 36 months. This pilot study indicates that the aforementioned 17 potential biomarkers, such as angiogenin, may hold great promise for both diagnosis and prognosis assessment of HFPEF.

## Introduction

Heart failure (HF), the most serious and the final outcome of all heart diseases, is a major and growing public health problem in the worldwide 1–4. Heart failure with preserved ejection fraction (HFPEF) not only represents half of the population of HF 5,6, but may lead to higher mortality, as compared with the HF with reduced left ventricular ejection fraction (LVEF) 7. Meanwhile, patients with an LVEF in the range of 41–49% represent an intermediate group, and this subset of HFPEF patients may either have persistently preserved LVEF or previously had HFREF 8,9. Notably, however, there exist relatively few strategies for early diagnosis and treatment of HFPEF. Therefore, finding more valuable biomarkers would give great impetus to accurate diagnosis and evaluation of HFPEF.

Cytokines, which can be produced by various types of cells (*e.g*. vascular wall cells and cardiac myocytes), are thought to play important roles in the pathophysiology and development of HF 10. Thus, these increased cytokines in systemic circulation may be potential candidates of biomarkers for HFPEF. Among the circulating cytokines, angiogenin is a potent inducer of neovascularization 11, which has been found to be associated with cardiovascular diseases such as chronic HF, coronary heart disease and cardiogenic shock 12–17, indicating that it may be a novel disease-specific biomarker.

The aims of the present study were to determine cytokine expression profile in the serum of patients with HFPEF and to explore the potential roles of angiogenin in both diagnosis and prognosis of the disease.

## Materials and methods

### Patients and controls

The definition of HFPEF is based on guidelines from the European Society of Cardiology 18. Meanwhile, a subset of HFPEF patients with LVEF ranging from 41 to 49%, which may also be defined as intermediate group, were also included in this study 8. Concretely, patients (aged >40 year-old) consecutively admitted to our hospital were eligible for enrolment if they had: (*i*) New York Heart Association (NYHA) functional class III to IV; (*ii*) LVEF of >40%, as documented by echocardiography and (*iii*) N-terminal pro-B-type natriuretic peptide (NT-proBNP) >1500 pg/ml. Patients were excluded if they: (*i*) had clinically significant myocardial infarction or angina pectoris; (*ii*) had implantable cardioverter defibrillator therapy or percutaneous coronary intervention, coronary bypass surgery or heart transplantation within 3 months; (*iii*) severe obstruction with hypertrophic obstructive cardiomyopathy; (*iv*) had severe diseases such as tumour, HIV infection, *etc*. Hypertensive patients and healthy individuals (without hypertension, diabetes mellitus, atrial fibrillation, *etc*.) were recruited as controls from the Department of Health Examination in our hospital.

The microarray cohort included three HFPEF patients, three hypertensive patients and three healthy individuals; the second validation cohort was composed of 16 HFPEF patients and 16 healthy individuals (without hypertension, diabetes mellitus, atrial fibrillation, *etc*.). Fasting whole blood were obtained from each participant and the sera were collected and stored at −80°C. All participants signed an informed written consent to participate in the study that was approved by Ethical Committee of Zhongshan Hospital, Fudan University, China.

### Protein quantitation and serum cytokines detection

Protein levels in the sera of three HFPEF patients, three hypertensive patients and three healthy controls were quantified with BCA Protein Assay Kit (KangChen, Shanghai, China). Subsequently, a wide array of 507 proteins (including cytokines, chemokines, adipokine, growth factors, angiogenic factors, proteases, soluble receptors, soluble adhesion molecules, *et*c.; Table S1) were detected with Human Cytokine Antibody Array Kit (RayBiotech, Norcross, GA, USA) according to the manufacturer’s instructions. Briefly, after blocking, membranes were incubated at room temperature (RT) for 2 hrs with 10-fold diluted sera. The membranes were washed and then incubated with biotin-conjugated antibodies at RT for 1 hr. The membranes were washed again and incubated with horseradish peroxidase-conjugated streptavidin at RT for 2 hrs, washed, and then developed. Finally, relative expression levels of the proteins were quantified by densitometry.

### Laboratory tests and serum angiogenin detection

Serum angiogenin levels were tested in 16 HFPEF patients and 16 healthy controls by enzyme-linked immunosorbent assay kit (R&D Systems, Minneapolis, MN, USA) according to the manufacturer’s instructions. Briefly, standard or sample was added into per well and incubated for 1 hr. Subsequently, wash buffer, conjugate, substrate solution and stop solution were added according to the instruction. Finally, we used a microplate reader to determine the optical density. Other biochemical tests were all performed with routine clinical auto-analyser assays in the Biochemistry Department of Zhongshan Hospital, including NT-proBNP, serum cholesterol, triglycerides, alanine aminotransferase, urea nitrogen, creatinine, *etc*.

### Follow-up

All patients were followed-up for 36 months by outpatient clinic attendance, telephone contact, or review of the medical notes. All-cause death was defined as adverse end-point.

### Power calculation

We were unaware of previous studies assessing the cross-sectional differences in angiogenin between HFPEF patients and healthy controls, nor the prognostic implications, to power our study. It had been previously reported that angiogenin levels in HFREF exceeded those of healthy controls 17, we therefore suggested a similar incremental increase in angiogenin levels in HFPEF patients. To achieve this similar increase at *P* < 0.05 and a 1-β power of 0.9, a minimum of 11 patients per group were required.

### Statistical analysis

Continuous variables were tested for normal distribution by Kolmogorov–Smirnov test and presented as mean ± SD or mean [95% confidence interval (CI)], as appropriate, while categorical variables as number of patients. Analyses were performed with SPSS version 16.0 (SPSS Inc., Chicago, IL, USA), STATA version 10.0 (StataCorp, College Station, Cary, TX, USA) and SAS version 9.2 (SAS Institute Inc., NC, USA). Comparisons between groups were performed by one-way anova followed by multiple comparisons performed with *post hoc* Bonferroni test and the significance of any differences between two groups were analysed by Student’s *t*-test. Categorical data were compared using the Chi-square test, and a Fisher’s exact test was performed, if relevant. The adjustment of different variables was performed with Logistic regression. Correlation between serum angiogenin and Lg(NT-proBNP) was assessed by Pearson’s correlation test. Receiver operator characteristic (ROC) curve was depicted by area under curve (AUC) with 95% CI. To compare the survival rate between the groups, Kaplan–Meier survival curves were calculated and tested by the log-rank test. Cluster analysis was performed with MultiExperiment Viewer version 4.2 (DFCI, Boston, MA, USA). A value of *P* < 0.05 was considered statistically significant.

## Results

### Demographical parameters

Table 1 shows characteristics of the nine female patients enrolled for protein microarray detection. None of the participants were suffering from diabetes mellitus or valvular heart disease, while both hypertension and atrial fibrillation were observed in all patients with HFPEF (LVEF >40%). Moreover, NT-proBNP was significantly increased in HFPEF patients. More detail clinical information regarding the HFPEF patients are shown in Table S2.

**Table 1:** Clinical characteristics of patients included in microarray detection

	Healthy control (1) (*n* = 3)	Hypertension (2) (*n* = 3)	HFPEF* (3) (*n* = 3)	*P*-value	
				(1) *versus* (3)	(2) *versus* (3)
Age (years)	69 ± 5	60 ± 2	73 ± 7	NS	<0.05
Sex (female, *n*)	3	3	3	–	–
Smoker (*n*)	0	0	0	–	–
Diabetes mellitus (*n*)	0	0	0	–	–
Hypertension (*n*)	0	3	3	–	–
Atrial fibrillation (*n*)	0	0	3	–	–
VHD (*n*)	0	0	0	–	–
LVEF (%)	63 ± 7	71 ± 6	57 ± 1	NS	NS
LAD (mm)	30 ± 4	36 ± 7	49 ± 2	<0.01	<0.05
LVESD (mm)	26 ± 5	26 ± 4	38 ± 7	NS	NS
LVEDD (mm)	41 ± 5	43 ± 3	55 ± 5	<0.05	<0.05
NT-proBNP (pg/ml)	61 ± 8	536 ± 371	2486 ± 924	<0.01	<0.01
TC (mmol/l)	4.99 ± 0.37	5.23 ± 0.47	5.08 ± 1.46	NS	NS
TG (mmol/l)	0.78 ± 0.27	0.92 ± 0.53	2.03 ± 0.78	NS	NS

* HFPEF patients with LVEF >40%.

HFPEF: heart failure with preserved ejection fraction; VHD: valvular heart disease; LVEF: left ventricular ejection fraction; LAD: left atrial diameter; LVESD: left ventricular end-systolic dimension; LVEDD: left ventricular end-diastolic dimension; NT-proBNP: N-terminal pro-B-type natriuretic peptide; TC: total cholesterol; TG: triglyceride; NS: not significant. Values are expressed as mean ± SD or as indicated.

Clinical characteristics of the 16 healthy individuals and 16 HFPEF patients (LVEF >40%) enrolled for serum angiogenin detection are shown in Table 2. The patients, most of who were accompanied by hypertension, diabetes mellitus, atrial fibrillation and even mild cardiac remodelling, were older than healthy controls. Moreover, to explore the difference in angiogenin level between the two subsets of HFPEF, the patients were divided into two subgroups according to LVEF (one group = 41–49% and the other ≥50%; Table 3). Specifically, all patients in the low-LVEF group, but not those in the higher LVEF group, showed various degrees of systolic dysfunction, as determined by echocardiographic analysis, suggesting these patients might previously be HFREF and the LVEF had been improved.

**Table 2:** Clinical characteristics of patients included in the study

	Healthy control (*n* = 16)	HFPEF* (*n* = 16)	*P*-value
Age (years)	68 ± 8	76 ± 4	0.001
Sex (female, *n*)	6	10	0.289
LVEF (%)	70 ± 4	55 ± 12	<0.001
LAD (mm)	34 ± 3	45 ± 6	<0.001
LVESD (mm)	26 ± 3	36 ± 8	<0.001
LVEDD (mm)	44 ± 4	52 ± 7	<0.001
NT-proBNP (pg/ml)	55 (27–93)	3377 (2178–3995)	<0.001
ALT (U/l)	15 (13–18)	19 (10–30)	0.30
BUN (μmol/l)	5.85 (5.13–6.73)	6.90 (5.63–8.18)	0.10
SCr (μmol/l)	67 (63–79)	90 (71–117)	0.01
TC (mmol/l)	4.94 ± 0.73	4.17 ± 0.99	0.018
TG (mmol/l)	1.11 (0.81–1.44)	1.17 (1.00–1.86)	0.27

* HFPEF patients with LVEF >40%.

HFPEF: heart failure with preserved ejection fraction; LVEF: left ventricular ejection fraction; LAD: left atrial diameter; LVESD: left ventricular end-systolic dimension; LVEDD: left ventricular end-diastolic dimension; NT-proBNP: N-terminal pro-B-type natriuretic peptide; ALT: alanine aminotransferase; BUN: blood urea nitrogen; SCr: serum creatinine; TC: total cholesterol; TG: triglyceride. Values are expressed as mean ± SD, mean (95% confidence interval) or as indicated.

**Table 3:** Clinical characteristics of patients included for subgroup analyses

	Healthy control (1) (*n* = 16)	HFPEF* (2) (*n* = 9)	HFPEF† (3) (*n* = 7)	*P*-value		
				(1) *versus* (2)	(1) *versus* (3)	(2) *versus* (3)
Age (years)	68 ± 8	77 ± 5	75 ± 3	0.003	0.021	0.634
Sex (female, *n*)	6	4	6	1.000	0.069	0.145
LVEF (%)	70 ± 4	64 ± 7	43 ± 2	0.006	0.000	0.000
LAD (mm)	34 ± 3	45 ± 5	45 ± 7	0.000	0.000	0.837
LVESD (mm)	26 ± 3	32 ± 7	40 ± 6	0.011	0.000	0.006
LVEDD (mm)	44 ± 4	49 ± 8	56 ± 5	0.017	0.000	0.033
NT-proBNP (pg/ml)	55 (27–93)	3530 (2874–3869)	2252 (2067–4038)	0.000	0.000	0.368
ALT (U/l)	15 (13–18)	22 (19–39)	13 (8–16)	0.020	0.383	0.068
BUN (μmol/l)	5.85 (5.13–6.73)	7.00 (6.30–8.00)	6.00 (4.40–8.30)	0.033	0.688	0.491
SCr (U/l)	67 (63–79)	114 (81–118)	79 (63–103)	0.006	0.242	0.223
TC (mmol/l)	4.94 ± 0.73	3.86 ± 0.73	4.56 ± 1.19	0.005	0.328	0.109
TG (mmol/l)	1.11 (0.81–1.44)	1.08 (0.91–1.42)	1.46 (1.10–2.23)	0.865	0.077	0.153

* HFPEF patients with LVEF ≥50%.

† HFPEF patients with LVEF = 41–49%.

HFPEF: heart failure with preserved ejection fraction; LVEF: left ventricular ejection fraction; LAD: left atrial diameter; LVESD, left ventricular end-systolic dimension; LVEDD: left ventricular end-diastolic dimension; NT-proBNP: N-terminal pro-B-type natriuretic peptide; ALT: alanine aminotransferase; BUN: blood urea nitrogen; SCr: serum creatinine; TC: total cholesterol; TG: triglyceride. Values are expressed as mean ± SD, mean (95% confidence interval) or as indicated.

### Analysis of antibody microarrays

A total of 507 known proteins (*e.g*. cytokines, chemokines, adipokine, growth factors, angiogenic factors, proteases, soluble receptors and soluble adhesion molecules) were measured in the sera of nine patients. The differences in protein expression among three groups are shown in Table S3 (all *P* < 0.05). The results showed that 59 proteins were up-regulated in HFPEF patients, as compared with healthy controls. More specifically, 11 of these proteins were increased by more than fivefold, including angiogenin, Activin A, Activin B, Artemin, CD80, tumour necrosis factor receptor superfamily members (TNFRSF13C and TNFRSF18), burkitt lymphoma receptor 1 (BLR-1), interleukin 15 receptor alpha (IL-15 R alpha), thrombopoietin (TPO) and thrombospondin-4. In addition, 17 proteins in the sera of HFPEF patients were significantly increased than that in patients with hypertension, while angiogenin was the only one that was increased by more than five times.

**Figure 1:**
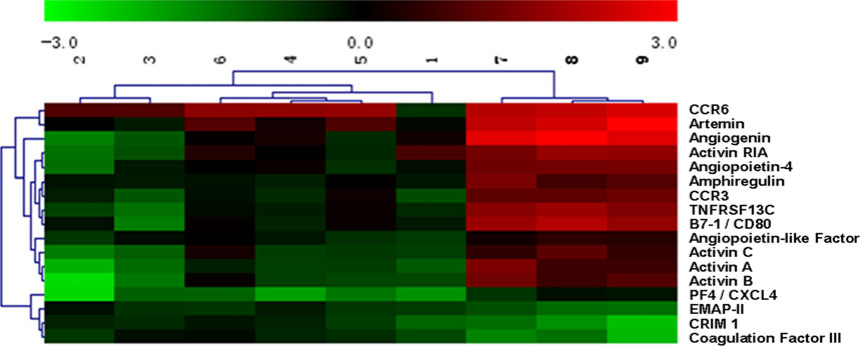
Heat map generated from protein microarray data reflecting protein expression values of the 17 proteins in all enrolled participants. Samples are arranged in columns, proteins in rows. Red shades, increased expression in heart failure with preserved ejection fraction (HFPEF) samples as compared to non-HFPEF samples (hypertensive patients and healthy controls); green shades, reduced expression; black, median expression. Samples are clustered into HFPEF and non-HFPEF categories as indicated by the first-order branches of the dendrogram (two black bars at the top; Clinical diagnosis of serum sample donor: 1–6, non-HFPEF patients; 7–9, HFPEF patients).

Furthermore, of the 507 proteins, 17 proteins were identified with significant difference in expression between HFPEF and non-HFPEF patients (both healthy individuals and hypertensive patients; Table 4). Concretely, 14 of these proteins were up-regulated in HFPEF patients, while three were down-regulated. Subsequently, all samples were arranged by the similarity in abundance of these 17 markers in the sera with an unsupervised clustering algorithm, which produced two main clusters that respectively contained HFPEF patients or non-HFPEF patients (Fig. 1), suggesting that the serum concentrations of many secreted proteins differ considerably between HFPEF patients and patients without HFPEF.

**Table 4:** Different cytokines levels in HFPEF patients compared with both hypertensive patients and healthy controls

Cytokines	HFPEF* *versus* hypertension		HFPEF* *versus* healthy control	
	Ratio	*P*-value	Ratio	*P*-value
Up-regulated				
Activin A	3.0506	0.0239	5.3430	0.0132
Activin B	2.7335	0.0141	5.5448	0.0043
Activin C	2.0881	0.0353	3.5598	0.0054
Activin RIA	3.0310	0.0029	3.4280	0.0133
Angiogenin	6.8233	0.0003	10.2767	0.0004
Angiopoietin-4	2.7800	0.0002	3.6353	0.0003
Angiopoietin-like factor	1.5721	0.0185	1.8272	0.0078
Amphiregulin	2.3934	0.0148	2.5769	0.0114
Artemin	3.7418	0.0172	6.9449	0.0094
B7-1/CD80	3.9195	0.0009	5.4942	0.0009
TNFRSF13C	3.3639	0.0007	5.4992	0.0004
CCR3	2.4836	0.0008	3.7077	0.0002
CCR6	1.5521	0.0112	3.3886	0.0034
PF4/CXCL4	2.1654	0.0084	2.5660	0.0113
Down-regulated				
Coagulation factor III	0.4074	0.0049	0.4633	0.0296
CRIM 1	0.4196	0.0082	0.4905	0.0466
EMAP-II	0.6462	0.0228	0.6202	0.0407

* HFPEF patients with LVEF >40%.

HFPEF: heart failure with preserved ejection fraction; ALK-2: activin receptor-like kinase-2; TNFRSF13C: tumour necrosis factor receptor superfamily member 13C; CCR3: CC chemokine receptor 3; CCR6: CC chemokine receptor 6; PF4: platelet factor 4; CXCL4: chemokine (C-X-C motif) ligand 4; CRIM 1: cysteine-rich motor neuron 1 protein; EMAP- II: endothelial monocyte-activating polypeptide II.

### Serum angiogenin level in HFPEF patients

**Figure 2:**
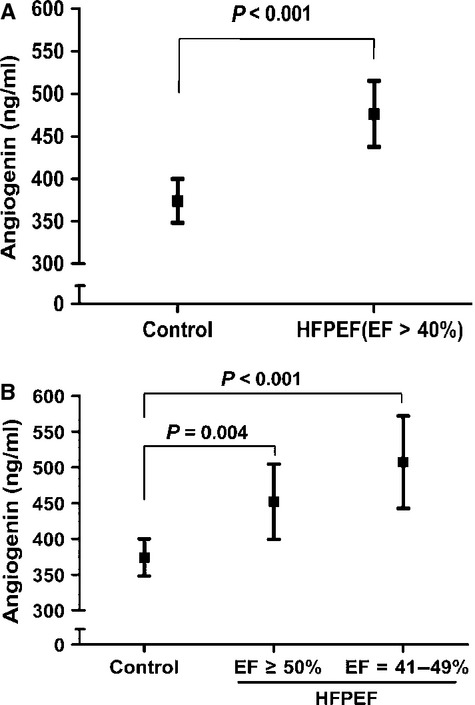
Circulating angiogenin levels in patients with heart failure with preserved ejection fraction (HFPEF) and healthy controls. (**A**) Serum angiogenin levels were increased in HFPEF patients with left ventricular ejection fraction (LVEF) >40% (*P* < 0.001), as compared with healthy controls. (**B**) Serum angiogenin levels were elevated in HFPEF patients with either LVEF = 41–49% (*P* < 0.001) or LVEF ≥50% (*P* = 0.004). Values indicated are mean (95% confidence interval).

According to the results of microarrays analyses, angiogenin was increased in HFPEF patients, as compared with both hypertensive patients and healthy controls, while significant difference was not observed between hypertensive patients and healthy controls (Table S3 and Table 4). To validate the elevated serum angiogenin level in HFPEF patients, a second set of serum samples from 16 HFPEF patients and 16 healthy controls were analysed. The results showed that the average angiogenin level was 103 ng/ml higher in HFPEF patients (477 ng/ml, 95% CI 438–515 ng/ml) than in healthy controls (374 ng/ml, 95% CI 348–400 ng/ml; *P* < 0.001; Fig. 2A), while the age-adjusted difference between the two groups remained statistically significant (*P* < 0.01). In addition, we found no impact of the HFPEF risk factors (including sex, hypertension, diabetes mellitus and atrial fibrillation) on angiogenin expression (Table S4), which was in accordance with the results of antibody microarrays analyses.

To further investigate the serum angiogenin levels in the above-mentioned two subsets of HFPEF, subgroup analyses were also performed. The results showed that the angiogenin levels were increased in HFPEF patients with either LVEF = 41–49% (508 ng/ml, 95% CI 433–572 ng/ml; *P* < 0.001) or LVEF ≥50% (452 ng/ml, 95% CI 400–505 ng/ml; *P* = 0.004), as compared with controls. Nevertheless, the mean level of angiogenin in patients with lower LVEF was 56 ng/ml increased than those with higher LVEF, but the difference was not statistically significant (Fig. 2B).

### Correlation between angiogenin and Lg(NT-proBNP)

**Figure 3:**
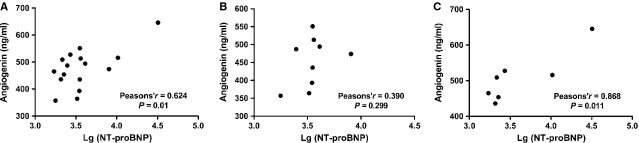
Correlation between serum angiogenin level and Lg(N-terminal pro-B-type natriuretic peptide, NT-proBNP) in patients with heart failure with preserved ejection fraction (HFPEF). (**A**) Angiogenin levels were positively correlated with Lg(NT-proBNP) in HFPEF patients with left ventricular ejection fraction (LVEF) >40% (*P* = 0.01). (**B**) No significant linear correlation was observed between angiogenin and Lg(NT-proBNP) in HFPEF patients with LVEF ≥50%. (**C**) Angiogenin levels were positively correlated with Lg(NT-proBNP) in HFPEF patients with LVEF = 41–49% (*P* = 0.01).

The results of Pearson’s correlation test showed that the serum angiogenin levels were positively correlated with Lg(NT-proBNP) not only in HFPEF patients with LVEF >40% (*r* = 0.62; *P* = 0.01; Fig. 3A), but also in a subset of HFPEF patients with LVEF = 41–49% (*r* = 0.87; *P* = 0.01; Fig. 3C). However, the same scene was not observed in patients with LVEF ≥50% (Fig. 3B).

### Angiogenin as a predictor of HFPEF

**Figure 4:**
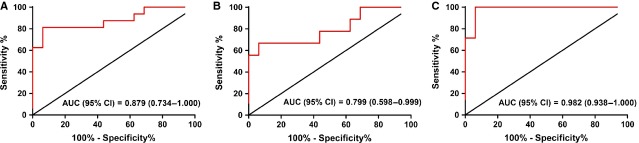
Receiver operator characteristic (ROC) curves of angiogenin for diagnosis of heart failure with preserved ejection fraction (HFPEF). (**A**) ROC curves of angiogenin for HFPEF patients with left ventricular ejection fraction (LVEF) >40% (*P* < 0.001). (**B**) ROC curves for HFPEF patients with LVEF ≥50% (*P* < 0.05). (**C**) ROC curves for HFPEF patients with LVEF = 41–49% (*P* < 0.001). Area under the curve (AUC) is shown for the performance of the angiogenin levels in discriminating HFPEF patients from healthy control. Values indicated are mean (95% confidence interval).

Receiver operator characteristic curves were used to evaluate the performance of angiogenin in HFPEF patients. The results showed that the mean AUC concerning the patients with LVEF >40% was 0.88 (95% CI 0.73–1.00; *P* < 0.001) with a sensitivity, specificity and cut-off point of 81%, 94% and 426 ng/ml (Fig. 4A), suggesting angiogenin may be a discriminator between these patients and healthy controls. Moreover, the results of logistic regression analyses showed that 10 ng/ml and 20 ng/ml up-regulation of serum angiogenin level was separately in correspondence to 37% and 87% increase in the risk of suffering from the disease.

In addition, the results of subgroup analyses showed that angiogenin may also distinguish HFPEF patients with either LVEF ≥50% (AUC: 0.80, 95% CI 0.60–1.00; *P* < 0.05; Fig. 4B) or LVEF = 41–49% (AUC: 0.98, 95% CI 0.94–1.00; *P* < 0.001; Fig. 4C) from healthy controls. More concretely, with a cut-off point of 426 ng/ml, the sensitivity and specificity for diagnosing the former population was 67% and 94%, which rose up to 100% and 94% for the later. However, we failed to distinguish the two subsets of the patients from each other by angiogenin.

### Clinical end-point and survival analyses

All the 16 patients enrolled were followed-up by 36 months and all-cause death was recorded in eight patients (50%) as the clinical end-point.

**Figure 5:**
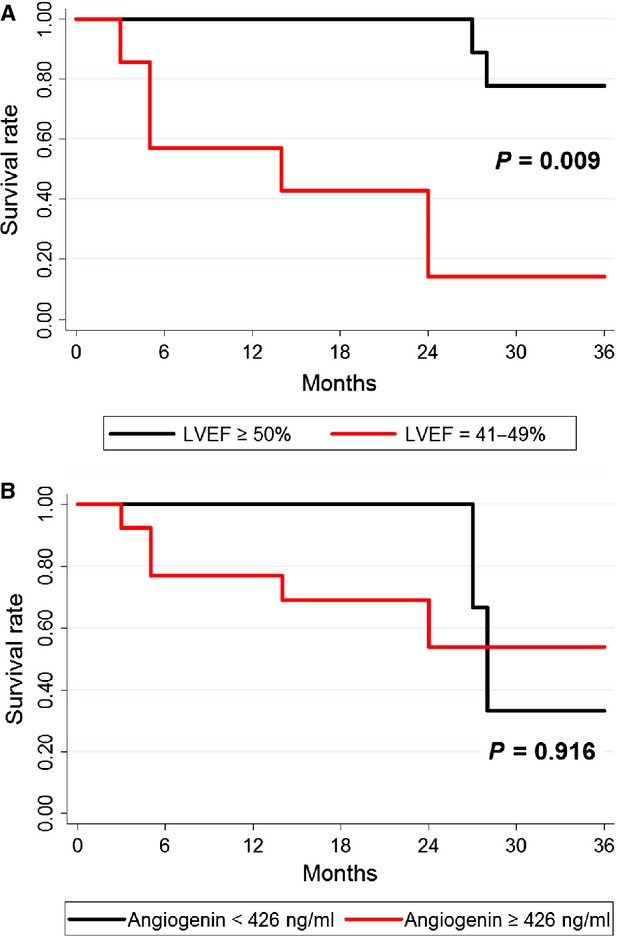
Kaplan–Meier curves for all-cause mortality in the follow-up duration of 36 months. (**A**) Heart failure with preserved ejection fraction (HFPEF) patients stratified according to baseline angiogenin levels (red line, ≥426 ng/ml; black line, <426 ng/ml). (**B**) HFPEF patients stratified according to baseline left ventricular ejection fraction (LVEF) levels (red line = 41–49%; black line = ≥50%).

The results of Cox regression analyses showed that low baseline LVEF (41–49%) was a significant predictor of adverse outcome (Hazard ratio: 9.55, 95% CI 1.77–51.42; *P* = 0.009). Kaplan–Meier survival curves showed that the prognosis seemed to be better in HFPEF patients with baseline LVEF ≥50% than those with baseline LVEF = 41–49% in 36 months (Fig. 5A). Although the survival rate in patients with low baseline angiogenin levels (<426 ng/ml) seemed to be higher within 24 months, we failed to find high baseline angiogenin levels (≥426 ng/ml) as a significant predictor of all-cause death in the longer term follow-up duration of 36 months (Fig. 5B), by using the cut-off value based on ROC curve analysis.

## Discussion

The high morbidity and mortality of HFPEF patients necessitate more effective strategies for optimal clinical management of the disease, including diagnosis, defining the disease state, assessing of individual risk profiles and setting up individual therapeutic strategies 5–7. In this study, we detected protein expression profile in the sera of patients with HFPEF by using antibody microarrays, and identified angiogenin as a potential biomarker for both diagnosis and prognosis of the disease.

Given that proteins are the primary effectors of cellular function and recent advances in proteomic technologies permit the evaluation of systematic changes in protein expression in response to intrinsic or extrinsic perturbations to the biological system, a great quantity of researches focused on the role of proteins in HF to outline both mechanisms and biomarkers of diseases 19–23. However, few studies have been performed concerning HFPEF patients with either LVEF ≥50% or LVEF = 41–49%, a subset of HFPEF that may previously had HFREF 8,9.

In this study, a wide array of 507 different proteins were firstly detected in the sera of nine female patients, including HFPEF patients with LVEF >40%, hypertensive patients and healthy controls, concerning that female and hypertension are considered as two of the underlying factors in HFPEF 24–27. The results showed that 17 proteins in HFPEF patients were significantly different from that in non-HFPEF patients, encompassing angiogenin, activin A, artemin, *etc*.

In the past decades, although numerous biomarkers, such as BNP, NT-proBNP, cardiac troponin T/I, interleukin family member ST2 and galectin-3, have emerged that might aid in the complex decision-making processes for diagnosis, evaluation and treatment of HF 8,28, multimarker strategy might be warranted in future because an ideal biomarker is required to have high specificity, sensitivity and reproducibility, little biovariability, and independence of demographical characteristics 29. Based on the above-mentioned 17 proteins, cluster analysis yielded two main clusters that obviously distinguished HFPEF patients from those without HFPEF, suggesting that detection of these proteins as a whole might serve as a novel potential strategy for the diagnosis of HFPEF. In addition, targeting these cytokines and receptors may offer new opportunities for therapeutic interventions.

Of the 17 distinctly expressed proteins, angiogenin, which has been considered as a potential prognostic and diagnostic biomarker in cardiovascular diseases 12–17, is one of the most potent angiogenic factors, with an essential role in vessel permeability, endothelial proliferation and vascular maturation 11.

To validate the up-regulation of angiogenin in HFPEF, it was further measured in a larger population. The results showed that serum angiogenin level was not only increased in HFPEF, regardless of the differences in clinical characteristics such as age, sex and diabetes, which is consistent with previous studies 30,31, but also positively correlated with Lg(NT-proBNP), which may provide prognostic information in these patients 32,33. Moreover, we found that angiogenin may be a discriminator between HFPEF patients and healthy controls, with a cut-off point of 426 ng/ml. Meanwhile, elevated angiogenin level may as well be a risk factor to predict HFPEF. These findings suggest that angiogenin may be a potential biomarker for both diagnosis and prognosis of the disease.

As mentioned above, not only HFPEF patients with LVEF ≥50% were studied in this study, but also those with both LVEF = 41–49% and impaired systolic function that exists despite preserved global LVEF 34. Likewise, the results showed that angiogenin levels were increased in these two subgroups of patients, meanwhile, it may act as a predictor for both of the two subsets of HFPEF. However, we failed in this study to distinguish the two subsets of the patients from each other by angiogenin, which may partially be accounted for by the small population enrolled.

In addition, by using the cut-off value based on ROC curve analysis, we found no difference in the survival rate between the patients with low angiogenin levels (<426 ng/ml) and those with high levels (≥426 ng/ml) within a follow-up during of 36 months, which, from our point of view, may also be explained by the small size of the study. Concretely, there were only three patients with an angiogenin level of less than 426 ng/ml and none of them died within 24 months, while all the six adverse events were observed in this period of time among the other 13 patients with high angiogenin levels. Furthermore, the prognosis seemed to be better in HFPEF patients with LVEF ≥50% than those with lower LVEF, while such a low survival rate (six died in seven) in HFPEF patient with LVEF = 41–49% observed in this study may be because of the severity of the patients enrolled, with the NYHA classification of III to IV and NT-proBNP >1500 pg/ml 35,36.

In conclusion, this study indicates that the serum angiogenin level, which might be positively correlated with Lg(NT-proBNP) in HFPEF, may not only be increased in HFPEF patients with both LVEF ≥50% and LVEF ranging from 41 to 49%, but may also be a predictor for the disease. Meanwhile, although we failed to give a final answer for how to evaluate the prognosis of HFPEF by angiogenin in this pilot study, it may be a potential biomarker for both diagnosis and prognosis of the disease. Moreover, HFPEF patients with LVEF ≥50% may survive longer than those with both LVEF = 41–49% and systolic dysfunction. In addition, the small size and low power of this study resulted in the undefined roles of angiogenin in HFPEF, which necessitate more large-scale studies to be performed to validate the diagnostic and prognostic utility of angiogenin.

## Supporting Information

Table S1 Proteins measured with cytokine antibody array.

Table S2 Clinical characteristics of three HFPEF patients included in microarray detection.

Table S3 Comparison of cytokines levels among different groups.

Table S4 Impact of the disease risk factors on angiogenin levels in HFPEF.
