# Supplementary material for: A pilot study of angiogenin in heart failure with preserved ejection fraction: a novel potential biomarker for diagnosis and prognosis?
Source: J Cell Mol Med. 2014 Aug 15;18(11):2189–97. doi: 10.1111/jcmm.12344 (PMC4224553; doi:10.1111/jcmm.12344)
Supplement: Table S1 — Proteins measured with cytokine antibody array. [file jcmm0018-2189-sd1.doc]

**Supplemental Table 1. Proteins measured with cytokine antibody array**

| **No.** | **Name** | **No.** | **Name** | **No.** | **Name** | **No.** | **Name** | **No.** | **Name** |
| --- | --- | --- | --- | --- | --- | --- | --- | --- | --- |
| 1 | 6Ckine | 103 | EDA-A2 | 205 | HRG-beta 1 | 307 | Insulin R | 409 | Progranulin |
| 2 | Activin A | 104 | EDAR | 206 | HVEM / TNFRSF14 | 308 | Insulysin / IDE | 410 | Prolactin |
| 3 | Activin B | 105 | EDG-1 | 207 | I-309 | 309 | IP-10 | 411 | P-selectin |
| 4 | Activin C | 106 | EGF | 208 | ICAM-1 | 310 | I-TAC / CXCL11 | 412 | RAGE |
| 5 | Activin RIA / ALK-2 | 107 | EGF R / ErbB1 | 209 | ICAM-2 | 311 | Kininostatin / kininogen | 413 | RANK / TNFRSF11A |
| 6 | Activin RIB / ALK-4 | 108 | EG-VEGF / PK1 | 210 | ICAM-3 | 312 | Kremen-1 | 414 | RANTES |
| 7 | Activin RII A/B | 109 | EMAP-II | 211 | ICAM 5 | 313 | Kremen-2 | 415 | RELM beta |
| 8 | Activin RIIA | 110 | ENA-78 | 212 | IFN-alpha / beta R1 | 314 | LAP (TGF-beta 1) | 416 | RELT / TNFRSF19L |
| 9 | Adiponectin / Acrp30 | 111 | Endocan | 213 | IFN-alpha / beta R2 | 315 | Latent TGF-beta bp1 | 417 | ROBO4 |
| 10 | AgRP | 112 | Endoglin / CD105 | 214 | IFN-beta | 316 | LBP | 418 | S100 A8/A9 |
| 11 | ALCAM | | 113 | | --- | | Endostatin | 215 | IFN-gamma | 317 | LECT2 | 419 | S100A10 |
| 12 | Angiogenin | 114 | EN-RAGE | 216 | IFN-gamma R1 | 318 | Lefty - A | 420 | SAA |
| 13 | Angiopoietin-1 | 115 | Eotaxin / CCL11 | 217 | IGFBP-1 | 319 | Leptin R | 421 | SCF |
| 14 | Angiopoietin-2 | 116 | Eotaxin-2 / MPIF-2 | 218 | IGFBP-2 | 320 | Leptin (OB) | 422 | SCF R/CD117 |
| 15 | Angiopoietin-4 | 117 | Eotaxin-3 / CCL26 | 219 | IGFBP-3 | 321 | LFA-1 alpha | 423 | SDF-1 / CXCL12 |
| 16 | Angiopoietin-like 1 | 118 | Epiregulin | 220 | IGFBP-4 | 322 | LIF | 424 | sFRP-1 |
| 17 | Angiopoietin-like 2 | 119 | ErbB2 | 221 | IGFBP-6 | 323 | LIF R alpha | 425 | sFRP-3 |
| 18 | Angiopoietin-like Factor | 120 | ErbB3 | 222 | IGFBP-rp1 / IGFBP-7 | 324 | LIGHT / TNFSF14 | 426 | sFRP-4 |
| 19 | Angiostatin | 121 | ErbB4 | 223 | IGF-I | 325 | Lipocalin-1 | 427 | sgp130 |
| 20 | APJ | 122 | Erythropoietin (EPO) | 224 | IGF-I R | 326 | LRP-1 | 428 | SIGIRR |
| 21 | AR (Amphiregulin) | 123 | E-Selectin | 225 | IGF-II | 327 | LRP-6 | 429 | Siglec-5/CD170 |
| 22 | APRIL | 124 | ETL | 226 | IGF-II R | 328 | L-Selectin (CD62L) | 430 | Siglec-9 |

**Supplemental Table 1. (Continued)**

| **No.** | **Name** | **No.** | **Name** | **No.** | **Name** | **No.** | **Name** | **No.** | **Name** |
| --- | --- | --- | --- | --- | --- | --- | --- | --- | --- |
| 23 | Artemin | 125 | FADD | 227 | IL-1 alpha | 329 | Luciferase | 431 | SLPI |
| 24 | Axl | 126 | FAM3B | 228 | IL-1 beta | 330 | Lymphotactin / XCL1 | 432 | Smad 1 |
| 25 | B7-1 (CD80) | 127 | Fas / TNFRSF6 | 229 | IL-1 F5 / FIL1delta | 331 | Lymphotoxin beta / TNFSF3 | 433 | Smad 4 |
| 26 | BAFF R / TNFRSF13C | 128 | Fas Ligand | 230 | IL-1 F6 / FIL1 epsilon | 332 | Lymphotoxin beta R / TNFRSF3 | 434 | Smad 5 |
| 27 | BCMA / TNFRSF17 | 129 | FGF Basic | 231 | IL-1 F7 / FIL1 zeta | 333 | MAC-1 | 435 | Smad 7 |
| 28 | BD-1 | 130 | FGF-BP | 232 | IL-1 F8 / FIL1 eta | 334 | MCP-1 | 436 | Smad 8 |
| 29 | BDNF | 131 | FGF R3 | 233 | IL-1 F9 / IL-1 H1 | 335 | MCP-2 | 437 | SMDF / NRG1Isoform |
| 30 | beta-Catenin | 132 | FGF R4 | 234 | IL-1 F10 / IL-1HY2 | 336 | MCP-3 | 438 | Soggy-1 |
| 31 | beta-Defensin 2 | 133 | FGF R5 | 235 | IL-1 R3 / IL-1 R AcP | 337 | MCP-4 / CCL13 | 439 | Sonic Hedgehog (Shh N-terminal) |
| 32 | beta-NGF | 134 | FGF-4 | 236 | IL-1 R4 /ST2 | 338 | M-CSF | 440 | SPARC |
| 33 | BIK | 135 | FGF-5 | 237 | IL-1 R6 / IL-1 Rrp2 | 339 | M-CSF R | 441 | Spinesin |
| 34 | BLC / BCA-1 / CXCL13 | 136 | FGF-6 | 238 | IL-1 R8 | 340 | MDC | 442 | TACI / TNFRSF13B |
| 35 | BMP-2 | 137 | FGF-7 / KGF | 239 | IL-1 R9 | 341 | MFG-E8 | 443 | Tarc |
| 36 | BMP-3 | 138 | FGF-8 | 240 | IL-1 ra | 342 | MFRP | 444 | TCCR / WSX-1 |
| 37 | BMP-3b / GDF-10 | 139 | FGF-9 | 241 | IL-1 RI | 343 | MIF | 445 | TECK / CCL25 |
| 38 | BMP-4 | 140 | FGF-10 / KGF-2 | 242 | IL-1 RII | 344 | MIG | 446 | TFPI |
| 39 | BMP-5 | 141 | FGF-11 | 243 | IL-2 | 345 | MIP-1a | 447 | TGF-alpha |
| 40 | BMP-6 | 142 | FGF-12 | 244 | IL-2 R alpha | 346 | MIP-1b | 448 | TGF-beta 1 |
| 41 | BMP-7 | 143 | FGF-13 1B | 245 | IL-2 R beta (CD122) | 347 | MIP-1d | 449 | TGF-beta 2 |
| 42 | BMP-8 | 144 | FGF-16 | 246 | IL-2 R gamma | 348 | MIP 2 | 450 | TGF-beta 3 |
| 43 | BMP-15 | 145 | FGF-17 | 247 | IL-3 | 349 | MIP-3 alpha | 451 | TGF-beta 5 |

**Supplemental Table 1. (Continued)**

| **No.** | **Name** | **No.** | **Name** | **No.** | **Name** | **No.** | **Name** | **No.** | **Name** |
| --- | --- | --- | --- | --- | --- | --- | --- | --- | --- |
| 44 | BMPR-IA / ALK-3 | 146 | FGF-18 | 248 | IL-3 R alpha | 350 | MIP-3 beta | 452 | TGF-beta RI / ALK-5 |
| 45 | BMPR-IB / ALK-6 | 147 | FGF-19 | 249 | IL-4 | 351 | MMP-1 | 453 | TGF-beta RII |
| 46 | BMPR-II | 148 | FGF-20 | 250 | IL-4 R | 352 | MMP-2 | 454 | TGF-beta RIIb |
| 47 | BTC | 149 | FGF-21 | 251 | IL-5 | 353 | MMP-3 | 455 | TGF-beta RIII |
| 48 | Cardiotrophin-1 / CT-1 | 150 | FGF-23 | 252 | IL-5 R alpha | 354 | MMP-7 | 456 | Thrombopoietin (TPO) |
| 49 | CCL14 / HCC-1 / HCC-3 | 151 | FLRG | 253 | IL-6 | 355 | MMP-8 | 457 | Thrombospondin (TSP) |
| 50 | CCL28 / VIC | 152 | Flt-3 Ligand | 254 | IL-6 R | 356 | MMP-9 | 458 | Thrombospondin-1 |
| 51 | CCR1 | 153 | Follistatin | 255 | IL-7 | 357 | MMP-10 | 459 | Thrombospondin-2 |
| 52 | CCR2 | 154 | Follistatin-like 1 | 256 | IL-7 R alpha | 358 | MMP-11 (Stromelysin-3) | 460 | Thrombospondin-4 |
| 53 | CCR3 | 155 | Fractalkine | 257 | IL-8 | 359 | MMP-12 | 461 | Thymopoietin (TP) |
| 54 | CCR4 | 156 | Frizzled-1 | 258 | IL-9 | 360 | MMP-13 | 462 | Tie-1 |
| 55 | CCR5 | 157 | Frizzled-3 | 259 | IL-10 | 361 | MMP-14 | 463 | Tie-2 |
| 56 | CCR6 | 158 | Frizzled-4 | 260 | IL-10 R alpha | 362 | MMP-15 | 464 | TIMP-1 |
| 57 | CCR7 | 159 | Frizzled-5 | 261 | IL-10 R beta | 363 | MMP-16 / MT3-MMP | 465 | TIMP-2 |
| 58 | CCR8 | 160 | Frizzled-6 | 262 | IL-11 | 364 | MMP-19 | 466 | TIMP-3 |
| 59 | CCR9 | 161 | Frizzled-7 | 263 | IL-12 p40 | 365 | MMP-20 | 467 | TIMP-4 |
| 60 | CD14 | 162 | Galectin-3 | 264 | IL-12 p70 | 366 | MMP-24 / MT5-MMP | 468 | TL1A / TNFSF15 |
| 61 | CD27 / TNFRSF7 | 163 | GASP-1 / WFIKKNRP | 265 | IL-12 R beta 1 | 367 | MMP-25 / MT6-MMP | 469 | TLR1 |
| 62 | CD30 / TNFRSF8 | 164 | GASP-2 / WFIKKN | 266 | IL-12 R beta 2 | 368 | MPIF-1 / CCL23 | 470 | TLR2 |
| 63 | CD30 Ligand / TNFSF8 | 165 | GCP-2 / CXCL6 | 267 | IL-13 | 369 | MSP alpha Chain | 471 | TLR3 |
| 64 | CD40 / TNFRSF5 | 166 | GCSF | 268 | IL-13 R alpha 1 | 370 | MSP beta-chain | 472 | TLR4 |

**Supplemental Table 1. (Continued)**

| **No.** | **Name** | **No.** | **Name** | **No.** | **Name** | **No.** | **Name** | **No.** | **Name** |
| --- | --- | --- | --- | --- | --- | --- | --- | --- | --- |
| 65 | CD40 Ligand / TNFSF5 (CD154) | 167 | G-CSF R / CD 114 | 269 | IL-13 R alpha 2 | 371 | NAP-2 | 473 | TMEFF1 / Tomoregulin-1 |
| 66 | CD 163 | 168 | GDF1 | 270 | IL-15 | 372 | NCAM-1 / CD56 | 474 | TMEFF2 |
| 67 | Cerberus 1 | 169 | GDF3 | 271 | IL-15 R alpha | 373 | Neuritin | 475 | TNF-alpha |
| 68 | Chem R23 | 170 | GDF5 | 272 | IL-16 | 374 | NeuroD1 | 476 | TNF-beta |
| 69 | Chordin-Like 1 | 171 | GDF8 | 273 | IL-17 | 375 | Neuropilin-2 | 477 | TNF RI / TNFRSF1A |
| 70 | Chordin-Like 2 | 172 | GDF9 | 274 | IL-17B | 376 | Neurturin | 478 | TNF RII / TNFRSF1B |
| 71 | Ck beta 8-1 / CCL23 | 173 | GDF11 | 275 | IL-17B R | 377 | NGF R | 479 | TRADD |
| 72 | CLC | 174 | GDF-15 | 276 | IL-17C | 378 | NOV / CCN3 | 480 | TRAIL / TNFSF10 |
| 73 | CNTF | 175 | GDNF | 277 | IL-17D | 379 | NRG1 Isoform GGF2 | 481 | TRAIL R1 / DR4 / TNFRSF10A |
| 74 | CNTF R alpha | 176 | GFR alpha-1 | 278 | IL-17E | 380 | NRG1-alpha / HRG1-alpha | 482 | TRAIL R2 / DR5 / TNFRSF10B |
| 75 | Coagulation Factor III / Tissue Factor | 177 | GFR alpha-2 | 279 | IL-17F | 381 | NRG1-beta1 / HRG1-beta1 | 483 | TRAIL R3 / TNFRSF10C |
| 76 | CRIM 1 | 178 | GFR alpha-3 | 280 | IL-17R | 382 | NRG2 | 484 | TRAIL R4 / TNFRSF10D |
| 77 | Cripto-1 | 179 | GFR alpha-4 | 281 | IL-17RC | 383 | NRG3 | 485 | Trance / TNFSF11 |
| 78 | CRTH-2 | 180 | GITR / TNFRF18 | 282 | IL-17RD | 384 | NT-3 | 486 | TREM-1 |
| 79 | Cryptic | 181 | GITR L / TNFSF18 L | 283 | IL-18 BPa | 385 | NT-4 | 487 | TROY / TNFRSF19 |
| 80 | CTACK / CCL27 | 182 | Glucagon | 284 | IL-18 R alpha (IL-1 R5) | 386 | Orexin A | 488 | TSG-6 |
| 81 | CTGF / CCN2 | 183 | Glut1 | 285 | IL-18 R beta (AcPL) | 387 | Orexin B | 489 | TSLP |
| 82 | CTLA-4 /CD152 | 184 | Glut2 | 286 | IL-19 | 388 | OSM | 490 | TWEAK / TNFSF12 |
| 83 | CV-2 / Crossveinless-2 | 185 | Glut3 | 287 | IL-20 | 389 | Osteoactivin / GPNMB | 491 | TWEAK R / TNFRSF12 |

**Supplemental Table 1. (Continued)**

| **No.** | **Name** | **No.** | **Name** | **No.** | **Name** | **No.** | **Name** | **No.** | **Name** |
| --- | --- | --- | --- | --- | --- | --- | --- | --- | --- |
| 84 | CXCL14 / BRAK | 186 | Glut5 | 288 | IL-20 R alpha | 390 | Osteocrin | 492 | Ubiquitin+1 |
| 85 | CXCL16 | 187 | Glypican 3 | 289 | IL-20 R beta | 391 | Osteoprotegerin / TNFRSF11B | 493 | uPA |
| 86 | CXCR1 / IL-8 RA | 188 | Glypican 5 | 290 | IL-21 | 392 | OX40 Ligand / TNFSF4 | 494 | uPAR |
| 87 | CXCR2 / IL-8 RB | 189 | GM-CSF | 291 | IL-21 R | 393 | PARC / CCL18 | 495 | Vasorin |
| 88 | CXCR3 | 190 | GM-CSF R alpha | 292 | IL-22 | 394 | PD-ECGF | 496 | VCAM-1/CD106 |
| 89 | CXCR4 (fusin) | 191 | Granzyme A | 293 | IL-22 BP | 395 | PDGF R alpha | 497 | VE-Cadherin |
| 90 | CXCR5 (BLR-1) | 192 | GREMLIN | 294 | IL-22 R | 396 | PDGF R beta | 498 | VEGF |
| 91 | CXCR6 | 193 | GRO | 295 | IL-23 | 397 | PDGF-AA | 499 | VEGF R2/KDR |
| 92 | D6 | 194 | GRO-a | 296 | IL-23 R | 398 | PDGF-AB | 500 | VEGF R3 |
| 93 | DAN | 195 | Growth Hormone (GH) | 297 | IL-24 | 399 | PDGF-BB | 501 | VEGF-B |
| 94 | DANCE | 196 | Growth Hormone R (GHR) | 298 | IL-26 | 400 | PDGF-C | 502 | VEGF-C |
| 95 | DcR3 / TNFRSF6B | 197 | HB-EGF | 299 | IL-27 | 401 | PDGF-D | 503 | VEGF-D |
| 96 | Decorin | 198 | HCC-4 / CCL16 | 300 | IL-28A | 402 | PECAM-1 (CD31) | 504 | VEGI / TNFSF15 |
| 97 | Dkk-1 | 199 | HCR / CRAM-A/B | 301 | IL-29 | 403 | Pentraxin3 / TSG-14 | 505 | WIF-1 |
| 98 | Dkk-3 | 200 | Hepassocin | 302 | IL-31 | 404 | Persephin | 506 | WISP-1 / CCN4 |
| 99 | Dkk-4 | 201 | Heregulin / NDF / GGF / Neuregulin | 303 | IL-31 RA | 405 | PF4 / CXCL4 | 507 | XEDAR |
| 100 | DR3 / TNFRSF25 | 202 | HGF | 304 | Inhibin A | 406 | PlGF |  |  |
| 101 | DR6 / TNFRSF21 | 203 | HGFR | 305 | Inhibin B | 407 | PLUNC |  |  |
| 102 | Dtk | 204 | HRG-alpha | 306 | Insulin | 408 | Pref-1 |  |  |
