# Supplementary material for: A pilot study of angiogenin in heart failure with preserved ejection fraction: a novel potential biomarker for diagnosis and prognosis?
Source: J Cell Mol Med. 2014 Aug 15;18(11):2189–97. doi: 10.1111/jcmm.12344 (PMC4224553; doi:10.1111/jcmm.12344)
Supplement: Table S2 — Clinical characteristics of three HFPEF patients included in microarray detection. [file jcmm0018-2189-sd2.doc]

**Supplemental Table 2. Clinical characteristics of three HFPEF patients included in microarray d**etection

|  | **Patient 1** | **Patient 2** | **Patient 3** |
| --- | --- | --- | --- |
| Age (years) | 81 | 73 | 67 |
| Sex | Female | Female | Female |
| Main symptoms |  |  |  |
| Dyspnea / Duration | Yes / 7 years | Yes / 1 month | Yes / 2 years |
| Cardiopalmus / Duration | Yes / 4 months | No | Yes / 2 years |
| Stethalgia / Duration | Yes / 4 months | No | Yes / 2 years |
| Unable to lie down flat | Yes | No | Yes |
| Cough / Expectoration | No | Yes | Yes |
| Hemoptysis | No | No | No |
| Night sweat | No | No | No |
| Vomiting / Haematemesis | No | No | No |
| Hematochezia | No | No | No |
| Sour regurgitation / Belching | No | No | No |
| Abdominal pain | No | No | No |
| Dysuresia | No | No | No |
| Dizziness / Tinnitus | No | No | No |
| Chilly | No | No | No |
| Physical examination |  |  |  |
| Body temperature (armpit, ℃) | 37.1 | 36.9 | 36.8 |
| Heart rate (beats/minute) | 81 | 78 | 95 |
| Respiratory rate (times/minute) | 19 | 20 | 20 |
| Systolic blood pressure (mmHg) | 132 | 150 | 128 |
| Diastolic blood pressure (mmHg) | 78 | 82 | 80 |
| Xanthochromia (skin or sclera) | No | No | No |
| Enlarged lymph nodes | No | No | No |
| Thoracic deformity | No | No | No |
| Moist rales / Field | Yes /  Both lower lung | Yes /  Right lower lung | Yes /  The whole lung |
| Dry rales / Field | No | No | Yes /  The whole lung |
| Enlarged heart size | Yes | Yes | Yes |
| Heart rhythm | Normal | Abnormal | Abnormal |
| Heart murmur | No | No | No |
| Lower extremities edema | Yes (both sides) | Yes (both sides) | Yes (both sides) |
| Abnormal neuropathological signs | No | No | No |
| Echocardiography |  |  |  |
| LVEF (%) | 67 | 46 | 57 |
| ARD (mm) | 35 | 37 | 37 |

**Supplemental Table 2. (Continued)**

|  | **Patient 1** | **Patient 2** | **Patient 3** |
| --- | --- | --- | --- |
| LVESD (mm) | 32 | 46 | 37 |
| LVEDD (mm) | 51 | 60 | 53 |
| LAD (mm) | 48 | 48 | 51 |
| IVS thickness (mm) | 12 | 9 | 12 |
| LVPWT (mm) | 11 | 9 | 11 |
| Laboratory measurements |  |  |  |
| NT-proBNP (pg/ml) | 2716 | 2154 | 3530 |
| CTnT (ng/ml) | 0.865 | 0.012 | -- |
| CK-MB (U/L) | 11 | -- | -- |
| FT3 (pmol/L) | 4.1 | -- | 4.4 |
| FT4 (pmol/L) | 17.3 | -- | 18.4 |
| TSH (uIU/ml) | 4.88 | -- | 3.76 |
| Total protein (g/L) | 73 | 81 | 70 |
| Albumin (g/L) | 38 | 45 | 42 |
| Globulin (g/L) | 35 | 36 | 23 |
| TB (umol/L) | 6.3 | 8.7 | 6.2 |
| CB (umol/L) | 2.2 | 2.9 | 1.0 |
| ALT (U/L) | 33 | 16 | 17 |
| AST (U/L) | 31 | 23 | 19 |
| ALP (U/L) | 52 | 91 | 48 |
| LDH (U/L) | 203 | 161 | 245 |
| Urea (umol/L) | 7.0 | 6.0 | 6.3 |
| Creatinine (umol/L) | 121 | 69 | 151 |
| Uric acid (umol/L) | 811 | 454 | 424 |
| Glucose (mmol/L) | 6.5 | 5.3 | 6.2 |
| TC (mmol/L) | 4.81 | 6.65 | 3.77 |
| Triglyceride (mmol/L) | 2.39 | 2.56 | 1.14 |
| LDL (mmol/L) | 2.61 | 4.60 | 2.51 |
| HDL (mmol/L) | 1.11 | 0.89 | 0.74 |
| RBC (×10^12^/L) | 3.63 | 5.00 | 3.31 |

**Supplemental Table 2. (Continued)**

|  | **Patient 1** | **Patient 2** | **Patient 3** |
| --- | --- | --- | --- |
| Hemoglobin (g/L) | 116 | 144 | 104 |
| Platelet (×10^9^/L) | 250 | 263 | 137 |
| WBC (×10^9^/L) | 6.3 | 8.3 | 5.5 |
| Neutrophil (%) | 41.6 | 57.4 | 64.8 |
| Lymphocyte (%) | 49.9 | 33.0 | 22.4 |
| Eosinophil (%) | 2.6 | 4.9 | 2.9 |
| Basophil (%) | 0.0 | 0.1 | 0.4 |
| Personal history |  |  |  |
| Smoking | No | No | No |
| Drinking | No | No | No |
| Atrial fibrillation / Duration | Yes / 2 years | Yes / 3 years | Yes / 2 years |
| Hypertension / Duration | Yes / 30 years | Yes / 3 years | Yes / 40 years |
| Diabetes mellitus | No | No | No |
| Operation | Appendectomy  (20 years ago) | No | No |
| Allergy | Penicillin | No | No |
| Blood transfusion | No | No | No |

HFPEFindicates heart failure with preserved ejection fraction; LVEF, left ventricular ejection fraction; ARD, Aortic root diameter; LVESD, left ventricular end-systolic dimension; LVEDD, left ventricular end-diastolic dimension; LAD, left atrial diameter; IVS, interventricular septal; LVPWT, left ventricular posterior wall thickness; NT-proBNP, N-terminal pro-B-type natriuretic peptide; CTnT, cardiac troponin T; CK-MB, creatine kinase-MB; FT3, free triiodothyronine; FT4, free thyroxine; TSH, Thyroid Stimulating Hormone; TB, total bilirubin; CB, conjugated bilirubin; ALT, alanine aminotransferase; AST, aspartate aminotransferase; ALP, alkaline phosphatase; LDH, lactate dehydrogenase; TC, total cholesterol; LDL, low density lipoprotein; HDL, high density lipoprotein; RBC, red blood cell; WBC, white blood cell.
