# Supplementary material for: A pilot study of angiogenin in heart failure with preserved ejection fraction: a novel potential biomarker for diagnosis and prognosis?
Source: J Cell Mol Med. 2014 Aug 15;18(11):2189–97. doi: 10.1111/jcmm.12344 (PMC4224553; doi:10.1111/jcmm.12344)
Supplement: Table S3 — Comparison of cytokines levels among different groups. [file jcmm0018-2189-sd3.doc]

**Supplemental Table 3. Comparison of cytokines levels among different groups**

|  | **HFPEF *vs.* Healthy control** | |  | **HFPEF *vs.* Hypertension** | |  | **Hypertension *vs.* Healthy control** | |
| --- | --- | --- | --- | --- | --- | --- | --- | --- |
|  | **Name** | ***P*-value** |  | **Name** | ***P*-value** |  | **Name** | ***P*-value** |
| Up-regulated |  |  |  |  |  |  |  |  |
| 1~2 folds | Activin RIIA | 0.0197 |  | Angiopoietin-1 | 0.0153 |  | 6Ckine | 0.0112 |
|  | Angiopoietin-2 | 0.0216 |  | Angiopoietin-like Factor | 0.0185 |  | Activin A | 0.0367 |
|  | Angiopoietin-like Factor | 0.0079 |  | CCR6 | 0.0112 |  | Artemin | 0.0395 |
|  | BDNF | 0.0235 |  |  |  |  | BAFF R / TNFRSF13C | 0.0495 |
|  | Glut5 | 0.0397 |  |  |  |  | BMP-2 | 0.0439 |
|  | IGFBP-6 | 0.0411 |  |  |  |  | BMP-3b / GDF-10 | 0.0445 |
|  | PECAM-1 /CD31 | 0.0306 |  |  |  |  | BMP-5 | 0.0005 |
|  | Thrombospondin (TSP) | 0.0068 |  |  |  |  | CD30 / TNFRSF8 | 0.0269 |
|  | TIMP-1 | 0.0472 |  |  |  |  | CD40 Ligand / TNFSF5 /CD154 | 0.0313 |
|  | TNF-beta | 0.0233 |  |  |  |  | Cerberus 1 | 0.0406 |
|  | TRANCE | 0.0404 |  |  |  |  | Chordin-Like 1 | 0.0005 |
|  |  |  |  |  |  |  | Galectin-3 | 0.0369 |
|  |  |  |  |  |  |  | IGFBP-6 | 0.0461 |
|  |  |  |  |  |  |  | IL-1 F6 / FIL1 epsilon | 0.028 |
|  |  |  |  |  |  |  | IL-1 R9 | 0.0176 |
|  |  |  |  |  |  |  | IL-13 R alpha 1 | 0.0169 |
|  |  |  |  |  |  |  | IL-17C | 0.0321 |
|  |  |  |  |  |  |  | IL-22 | 0.0022 |
|  |  |  |  |  |  |  | MCP-4 / CCL13 | 0.044 |
|  |  |  |  |  |  |  | M-CSF R | 0.0249 |
|  |  |  |  |  |  |  | OSM | 0.0085 |

**Supplemental Table 3. (Continued)**

|  | **HFPEF *vs.* Healthy control** | |  | **HFPEF *vs.* Hypertension** | |  | **Hypertension *vs.* Healthy control** | |
| --- | --- | --- | --- | --- | --- | --- | --- | --- |
|  | **Name** | ***P*-value** |  | **Name** | ***P*-value** |  | **Name** | ***P*-value** |
|  |  |  |  |  |  |  | Osteoactivin / GPNMB | 0.0017 |
|  |  |  |  |  |  |  | Osteoprotegerin / TNFRSF11B | 0.0413 |
|  |  |  |  |  |  |  | RELT / TNFRSF19L | 0.0405 |
|  |  |  |  |  |  |  | Smad 5 | 0.0422 |
|  |  |  |  |  |  |  | TIMP-1 | 0.0306 |
| 2~3 folds | AR (Amphiregulin) | 0.0114 |  | Activin B | 0.0141 |  | BMPR-II | 0.0471 |
|  | BMPR-IA / ALK-3 | 0.0466 |  | Activin C | 0.0353 |  | CCR6 | 0.0133 |
|  | BMPR-II | 0.0314 |  | Angiopoietin-4 | 0.0002 |  | Chem R23 | 0.0389 |
|  | CCR2 | 0.0387 |  | AR (Amphiregulin) | 0.0148 |  | CNTF R alpha | 0.0407 |
|  | IL-16 | 0.0472 |  | CCR3 | 0.0008 |  | CXCR6 | 0.0246 |
|  | Kremen-2 | 0.0418 |  | LBP | 0.0334 |  | DAN | 0.0242 |
|  | MSP beta-chain | 0.0087 |  | PF4 / CXCL4 | 0.0084 |  | EDG-1 | 0.02 |
|  | PF4 / CXCL4 | 0.0113 |  |  |  |  | ErbB2 | 0.0284 |
|  | Smad 1 | 0.0022 |  |  |  |  | FGF Basic | 0.0195 |
|  | Smad 5 | 0.0056 |  |  |  |  | FGF-10 / KGF-2 | 0.0373 |
|  | Tie-1 | 0.0343 |  |  |  |  | FGF-11 | 0.0186 |
|  | TSG-6 | 0.0413 |  |  |  |  | GITR / TNFRF18 | 0.0064 |
|  | VEGF | 0.0079 |  |  |  |  | Growth Hormone R (GHR) | 0.0382 |
|  | VEGF-B | 0.0181 |  |  |  |  | IGFBP-2 | 0.0309 |
|  |  |  |  |  |  |  | IL-3 R alpha | 0.0118 |
|  |  |  |  |  |  |  | IL-17B | 0.0429 |
|  |  |  |  |  |  |  | IL-17B R | 0.0483 |

**Supplemental Table 3. (Continued)**

|  | **HFPEF *vs.* Healthy control** | |  | **HFPEF *vs.* Hypertension** | |  | **Hypertension *vs.* Healthy control** | |
| --- | --- | --- | --- | --- | --- | --- | --- | --- |
|  | **Name** | ***P*-value** |  | **Name** | ***P*-value** |  | **Name** | ***P*-value** |
|  |  |  |  |  |  |  | IL-17D | 0.0476 |
|  |  |  |  |  |  |  | IL-24 | 0.0245 |
|  |  |  |  |  |  |  | IL-26 | 0.0279 |
|  |  |  |  |  |  |  | Inhibin B | 0.0158 |
|  |  |  |  |  |  |  | LIF | 0.0032 |
|  |  |  |  |  |  |  | LIF R alpha | 0.0341 |
|  |  |  |  |  |  |  | Lymphotoxin beta / TNFSF3 | 0.0084 |
|  |  |  |  |  |  |  | MCP-3 | 0.0499 |
|  |  |  |  |  |  |  | MMP-2 | 0.0015 |
|  |  |  |  |  |  |  | MSP alpha Chain | 0.0315 |
|  |  |  |  |  |  |  | MSP beta-chain | 0.0397 |
|  |  |  |  |  |  |  | Neuritin | 0.0393 |
|  |  |  |  |  |  |  | PDGF R beta | 0.0136 |
|  |  |  |  |  |  |  | TACI / TNFRSF13B | 0.0377 |
|  |  |  |  |  |  |  | VEGF-B | 0.0034 |
| 3~4 folds | 6Ckine | 0.0372 |  | Activin A | 0.0239 |  | CD40 / TNFRSF5 | 0.0042 |
|  | Activin C | 0.0054 |  | Activin RIA / ALK-2 | 0.0029 |  | CD 163 | 0.0127 |
|  | Activin RIA / ALK-2 | 0.0133 |  | Artemin | 0.0172 |  | CXCR4 (fusin) | 0.0038 |
|  | ALCAM | 0.0401 |  | Axl | 0.0035 |  | CXCR5 /BLR-1 | 0.0203 |
|  | Angiopoietin-4 | 0.0003 |  | B7-1 /CD80 | 0.0009 |  | IGFBP-3 | 0.0098 |
|  | CCR1 | 0.0373 |  | BAFF R / TNFRSF13C | 0.0007 |  | IL-1 F5 / FIL1delta | 0.0036 |
|  | CCR3 | 0.0002 |  |  |  |  | IL-3 | 0.0024 |

**Supplemental Table 3. (Continued)**

|  | **HFPEF *vs.* Healthy control** | |  | **HFPEF *vs.* Hypertension** | |  | **Hypertension *vs.* Healthy control** | |
| --- | --- | --- | --- | --- | --- | --- | --- | --- |
|  | **Name** | ***P*-value** |  | **Name** | ***P*-value** |  | **Name** | ***P*-value** |
|  | CCR6 | 0.0034 |  |  |  |  | IL-8 | 0.0147 |
|  | ErbB2 | 0.0366 |  |  |  |  | IL-19 | 0.0096 |
|  | FGF Basic | 0.0355 |  |  |  |  | MMP-10 | 0.0112 |
|  | IGFBP-rp1 / IGFBP-7 | 0.0348 |  |  |  |  | Orexin A | 0.0035 |
|  | IL-8 | 0.0465 |  |  |  |  | PDGF-AA | 0.0145 |
|  | IL-17B | 0.0381 |  |  |  |  | PDGF-AB | 0.0037 |
|  | IL-19 | 0.0379 |  |  |  |  | PDGF-BB | 0.0189 |
|  | IL-24 | 0.0412 |  |  |  |  | sgp130 | 0.0303 |
|  | Tie-2 | 0.0177 |  |  |  |  | Smad 4 | 0.0162 |
|  | TIMP-2 | 0.0007 |  |  |  |  | TRAIL R4 / TNFRSF10D | 0.0496 |
|  | TWEAK / TNFSF12 | 0.0152 |  |  |  |  |  |  |
| 4~5 folds | IL-1 F5 / FIL1delta | 0.0249 |  |  |  |  | IL-15 R alpha | 0.0229 |
|  | MMP-10 | 0.0455 |  |  |  |  | MMP-8 | 0.0038 |
|  | Neuritin | 0.0322 |  |  |  |  | Siglec-5/CD170 | 0.0487 |
|  | Orexin A | 0.0301 |  |  |  |  | TGF-beta 2 | 0.0126 |
|  | TRAIL R4 / TNFRSF10D | 0.0055 |  |  |  |  | Thrombopoietin (TPO) | 0.0376 |
| 5~10 folds | Activin A | 0.0132 |  | Angiogenin | 0.0003 |  |  |  |
|  | Activin B | 0.0043 |  |  |  |  |  |  |
|  | Artemin | 0.0094 |  |  |  |  |  |  |
|  | B7-1 /CD80 | 0.0009 |  |  |  |  |  |  |
|  | BAFF R / TNFRSF13C | 0.0004 |  |  |  |  |  |  |
|  | CXCR5 /BLR-1 | 0.0235 |  |  |  |  |  |  |

**Supplemental Table 3. (Continued)**

|  | **HFPEF *vs.* Healthy control** | |  | **HFPEF *vs.* Hypertension** | |  | **Hypertension *vs.* Healthy control** | |
| --- | --- | --- | --- | --- | --- | --- | --- | --- |
|  | **Name** | ***P*-value** |  | **Name** | ***P*-value** |  | **Name** | ***P*-value** |
|  | GITR / TNFRSF18 | 0.0442 |  |  |  |  |  |  |
|  | IL-15 R alpha | 0.0255 |  |  |  |  |  |  |
|  | Thrombopoietin (TPO) | 0.0124 |  |  |  |  |  |  |
|  | Thrombospondin-4 | 0.0368 |  |  |  |  |  |  |
| >10 folds | Angiogenin | 0.0004 |  |  |  |  |  |  |
|  |  |  |  |  |  |  |  |  |
| Down-regulated |  |  |  |  |  |  |  |  |
| 1~2 folds | EMAP-II | 0.0407 |  | BLC / BCA-1 / CXCL13 | 0.0246 |  | IL-1 F10 / IL-1HY2 | 0.0475 |
|  | IL-13 | 0.0292 |  | BMP-3b / GDF-10 | 0.0232 |  |  |  |
|  |  |  |  | BMP-5 | 0.0031 |  |  |  |
|  |  |  |  | Chem R23 | 0.0109 |  |  |  |
|  |  |  |  | Chordin-Like 1 | 0.0042 |  |  |  |
|  |  |  |  | Cripto-1 | 0.0258 |  |  |  |
|  |  |  |  | EMAP-II | 0.0228 |  |  |  |
|  |  |  |  | GCP-2 / CXCL6 | 0.0095 |  |  |  |
|  |  |  |  | IL-3 R alpha | 0.0467 |  |  |  |
| 2~3 folds | Coagulation Factor III | 0.0296 |  | Coagulation Factor III | 0.0049 |  |  |  |
|  | CRIM 1 | 0.0466 |  | CRIM 1 | 0.0082 |  |  |  |
|  |  |  |  | DR6 / TNFRSF21 | 0.0015 |  |  |  |
|  |  |  |  | EDA-A2 | 0.0424 |  |  |  |
|  |  |  |  | EGF R / ErbB1 | 0.0015 |  |  |  |

HFPEF indicates heart failure with preserved ejection fraction.
