# Supplementary material for: A pilot study of angiogenin in heart failure with preserved ejection fraction: a novel potential biomarker for diagnosis and prognosis?
Source: J Cell Mol Med. 2014 Aug 15;18(11):2189–97. doi: 10.1111/jcmm.12344 (PMC4224553; doi:10.1111/jcmm.12344)
Supplement: Table S4 — Impact of the disease risk factors on angiogenin levels in HFPEF. [file jcmm0018-2189-sd4.doc]

**Supplemental Table 4. Impact of the disease risk factors on angiogenin levels in HFPEF**

|  | **Angiogenin (ng/ml)** | |  |
| --- | --- | --- | --- |
|  | **Yes** | **No** | ***P*-value** |
| Sex (male) | 446 (331 - 561) | 495 (345 - 645) | 0.201 |
| Hypertension | 484 (350 - 618) | 364* | 0.113 |
| Atrial fibrillation | 456 (324 - 589) | 510 (365 - 655) | 0.161 |
| Diabetes mellitus | 469 (301 - 637) | 484 (363 - 604) | 0.697 |

HFPEF indicates heart failure with preserved ejection fraction. Values are expressed as mean (95% confidence interval). *Only one patient enrolled was not accompanied by hypertension.
